# Lead, Diabetes, Hypertension, and Renal Function: The Normative Aging Study

**DOI:** 10.1289/ehp.7024

**Published:** 2004-06-03

**Authors:** Shirng-Wern Tsaih, Susan Korrick, Joel Schwartz, Chitra Amarasiriwardena, Antonio Aro, David Sparrow, Howard Hu

**Affiliations:** ^1^Occupational Health Program, Department of Environmental Health, Harvard School of Public Health, Boston, Massachusetts, USA; ^2^Channing Laboratory, Department of Medicine, Brigham and Women’s Hospital, and Harvard Medical School, Boston, Massachusetts, USA; ^3^Environmental Epidemiology Program, Department of Environmental Health, Harvard School of Public Health, Boston, Massachusetts, USA; ^4^The Normative Aging Study, Department of Veterans Affairs Medical Center, Boston, Massachusetts, USA

**Keywords:** blood lead, bone lead, diabetes, hypertension, kidney function, serum creatinine

## Abstract

In this prospective study, we examined changes in renal function during 6 years of follow-up in relation to baseline lead levels, diabetes, and hypertension among 448 middle-age and elderly men, a subsample of the Normative Aging Study. Lead levels were generally low at baseline, with mean blood lead, patella lead, and tibia lead values of 6.5 μg/dL, 32.4 μg/g, and 21.5 μg/g, respectively. Six percent and 26% of subjects had diabetes and hypertension at baseline, respectively. In multivariate-adjusted regression analyses, longitudinal increases in serum creatinine (SCr) were associated with higher baseline lead levels but these associations were not statistically significant. However, we observed significant interactions of blood lead and tibia lead with diabetes in predicting annual change in SCr. For example, increasing the tibia lead level from the midpoints of the lowest to the highest quartiles (9–34 μg/g) was associated with an increase in the rate of rise in SCr that was 17.6-fold greater in diabetics than in nondiabetics (1.08 mg/dL/10 years vs. 0.062 mg/dL/10 years; *p* < 0.01). We also observed significant interactions of blood lead and tibia lead with diabetes in relation to baseline SCr levels (tibia lead only) and follow-up SCr levels. A significant interaction of tibia lead with hypertensive status in predicting annual change in SCr was also observed. We conclude that longitudinal decline of renal function among middle-age and elderly individuals appears to depend on both long-term lead stores and circulating lead, with an effect that is most pronounced among diabetics and hypertensives, subjects who likely represent particularly susceptible groups.

An association between lead poisoning and renal disease in humans has been recognized for more than a century (Wedeen et al. 1975). Numerous epidemiologic studies, mortality studies, and experimental studies in animals have reported lead nephrotoxicity at high levels of exposure; however, studies on the action of lead on renal function at lower levels of chronic exposure have produced a mixed pattern of findings. Most of the studies found no significant association between low-level lead exposure and renal dysfunction. To date, only a few cross-sectional studies (Payton et al. 1994; Staessen et al. 1990, 1992) and one longitudinal study (Kim et al. 1996) have reported a significant association between elevated blood lead levels and reduced renal function measured by serum creatinine (SCr) or creatinine clearance in members of the general population. In addition, a recent randomized trial among individuals with elevated environmental lead exposure demonstrating improved creatinine clearance in those receiving chelation therapy provides evidence of lead’s effect on the kidney (and its potential reversibility) at community levels of exposure (Lin et al. 2003).

Blood lead, which mostly reflects relatively recent exposure, is an inadequate measure of total body burden of lead, which may explain why most of the previous observational studies failed to find a significant association between low-level lead exposure and renal function impairment. Compared with concurrent blood lead, bone lead, which comprises > 95% of adult body lead burden and has a biologic half-life ranging from years to decades, is a better biologic marker for studying chronic toxicity of accumulated exposure and lead burden (Gonzalez-Cossio et al. 1997; Hu et al. 1996; Korrick et al. 1999). In addition, bone lead also serves as an endogenous source of lead exposure for individuals with increased bone turnover (Silbergeld 1991; Silbergeld et al. 1988). Therefore, bone lead may be a risk factor for impaired renal function either by serving as either a dosimeter of cumulative exposure of the kidney to lead or a measure of the major endogenous source of blood lead that, in turn, may affect the kidney.

Given that an increase in bone resorption is a characteristic of aging in both men and women, aging-associated release of bone lead into the circulation is a potentially important source of soft-tissue lead exposure and toxicity. Another factor associated with aging that may increase the nephrotoxicity of lead is diabetes. The more prevalent form, type 2 diabetes, affects approximately 10% or more of the general population (with substantially higher rates at ≥ 55 years of age) (Ford 2001) and is well known as an independent predictor of accelerated decline in kidney function. A third factor associated with aging that may also increase the nephrotoxicity of lead is hypertension.

In the present study, we used data from a cohort of middle-age and elderly men who had no previous known heavy lead exposure to examine the effects of low-level bone and blood lead levels on renal function. We also examined the potential modifying effect of diabetes and hypertension on these relationships.

## Materials and Methods

### Study population.

Study participants were from the Normative Aging Study (NAS), a longitudinal study of aging established by the Veterans Administration in 1961 (Bell et al. 1972). The study cohort initially consisted of 2,280 men from the Greater Boston area who were 21–80 years of age on enrollment. All participants were free of known chronic medical conditions at enrollment; men with any history of cancer, asthma, sinusitis, bronchitis, diabetes, gout, or peptic ulcer were excluded, as were those with a systolic blood pressure of > 140 mmHg or a diastolic blood pressure of > 90 mmHg. Since their enrollment in 1961–1968, participants have been reevaluated at 3- to 5-year intervals by a detailed core examination including collection of medical history information, routine physical examinations, laboratory tests, and questionnaires. The mean of blood pressure measurements in the left and right arms was used as each participant’s systolic and diastolic blood pressure. For the present study, “hypertensive” was defined as systolic blood pressure ≥ 160, or diastolic blood pressure ≥ 95 mmHg, or a physician’s diagnosis of hypertension with use of antihypertensive medication. The diagnosis of diabetes was based on clinical data from the study core examination; specifically, participants were classified as diabetic if they *a*) used oral hypoglycemic drugs, *b*) used insulin, or *c*) reported a physician’s diagnosis of diabetes whether or not they used diabetic drugs for treatment.

A blood sample for lead analysis has been collected at each NAS visit since July 1988. Beginning in August 1991, NAS participants were recruited for a substudy of K X-ray fluorescence (KXRF) bone lead measurement. Subjects included in the present investigation were those who participated in the KXRF bone lead substudy with concurrent blood lead, SCr, body mass index (BMI), alcohol intake, and blood pressure data and a follow-up measurement of SCr at least 4 years later.

All research performed in the present study was approved by the Human Research Committees of Brigham and Women’s Hospital and the Department of Veterans Affairs Outpatient Clinic.

### Measurements.

Bone lead was measured in each subject’s midtibia shaft and patella with a KXRF instrument (ABIOMED, Inc., Danvers, MA). The tibia and patella have been targeted for bone lead research because they consist mainly of cortical and trabecular bone, respectively. A technical description and the validity specifications of this instrument have been published elsewhere (Burger et al. 1990; Hu et al. 1990). The KXRF instrument provides an unbiased estimate of bone lead levels (normalized for bone mineral content as micrograms of lead per gram of bone mineral) and an estimate of the uncertainty associated with each measurement.

Whole-blood samples were obtained and analyzed for lead by graphite furnace atomic absorption with Zeeman background correction (ESA Laboratories, Chelmsford, MA). Values below the minimum detection limit of 1 μg/dL were coded as 0. The instrument was calibrated with National Institute of Standards and Technology Standard Reference Material (NIST SRM 955a, lead in blood) after every 20 samples. Ten percent of samples were run in duplicate; at least 10% of the samples were controls, and 10% were blanks. In tests on reference samples from the Centers for Disease Control and Prevention (Atlanta, GA), precision [coefficient of variation (CV)] ranged from 8% for concentrations 10–30 μg/dL to 1% for higher concentrations. Compared with an NIST target of 5.7 μg/dL, 24 measurements by this method gave a mean ± SD of 5.3 ± 1.23 μg/dL.

SCr concentration was determined by a computerized automatic analyzer [Technicon SAM models (Technicon Corp., Tarrytown, NY) from 1979 to 1993; Boehringer Mannheim/Hitachi 747 analyzer (Boehringer-Mannheim Corp, Indianapolis, IN) from 1993 and on] at each examination. The analyzer measures creatinine based on the Jaffe procedure (Jaffe 1886) and demonstrated excellent reproducibility. This method of analysis has intraassay CVs of 1.3% at 1.2 mg/dL and interassay CVs of 3.3% at 1.1 mg/dL.

### Statistical methods.

We used chi-square analysis and Student’s *t*-test to compare participants included in the analysis with eligible nonparticipants. Because many of the variables had skewed distributions, we used the nonparametric Wilcoxon signed-rank test for continuous variables to compare their distributions between baseline and follow-up visit. The main outcome of interest, annual change in SCr (milligrams per deciliter per year) was defined as (follow-up SCr – baseline SCr)/years of follow-up.

We used multiple linear regression analyses to determine the associations between baseline lead biomarkers (blood lead, patella lead, and tibia lead) and annual change in SCr. Because lead levels in blood and bone were skewed toward the upper end, we used lead biomarkers in the natural log scale to improve stability over the whole range of lead levels. The following variables at baseline were considered for possible inclusion in the models: age, BMI, baseline SCr, diabetic status, hypertensive status, smoking history [smoking status (ever/never) and cumulative smoking in pack years], alcohol consumption, and use of analgesic medication and diuretic medication. Alcohol consumption was analyzed both as a continuous variable (grams per day) and as a categorical variable: nondrinkers, light to moderate drinkers (< 20 g/day), and heavy drinkers (≥ 20 g/day).

To examine the modifying effect of diabetes on the nephrotoxicity of lead, we constructed models of the hypothesized interaction of lead with diabetes as follows: Annual change in SCr = intercept + β_1_(*I*_D1_) + β_2_(*I*_D0_ × mean-centered lead) + β_3_ (*I*_D1_ × mean-centered lead) + (other covariates), where *I*_D0_ = 1 if nondiabetes (reference group), 0 otherwise; *I*_D1_ = 1 if diabetes, 0 otherwise. We used this model to get the slopes for the two groups and their statistical significance. We constructed a second multiple regression model containing all main effects and a two-way interaction between diabetic status and natural-log–transformed baseline lead bio-markers. The model is expressed as annual change in SCr = intercept + β_1_(*I*_D1_) + β_2_(mean-centered lead) + β_3_ (*I*_D1_ × mean-centered lead) + (other covariates), where *I*_D1_ = 1 if diabetes, 0 otherwise. The second model was to do the statistical test of the interaction. If β_3_ differs significantly from zero, then diabetes is a significant effect modifier. The inclusion of specific covariates in the final multiple linear regression models was based on statistical and biologic considerations. To minimize the possibility of reverse causation, we repeated the analyses of annual change in SCr after excluding subjects with a high SCr at baseline, as defined by a value > 1.5 mg/dL. In addition, we examined the cross-sectional associations of baseline lead biomarkers with SCr measured at baseline and follow-up visits. The same set of confounders was considered for possible inclusion in the cross-sectional analyses of SCr.

We used the same approach to examine the modifying effect of hypertension on the nephrotoxicity of lead. Analyses were conducted using the Statistical Analysis System (Unix SAS version 8.2; SAS Institute, Cary, NC).

## Results

An initial group of 707 NAS subjects who participated in the KXRF substudy between 1991 and 1995 and who had complete data on lead biomarkers, SCr, BMI, alcohol intake, medication use history, and diagnoses and blood pressure measurements were identified as eligible study subjects at baseline. Among them, 448 subjects had a follow-up measurement of SCr at, on average, 6 years later (range, 4–8). Selected characteristics of the 448 subjects at baseline and at follow-up are shown in Table 1. No significant differences were found with respect to the distributions of age, BMI, alcohol consumption, smoking status, diabetic status, hypertensive status, baseline SCr, and blood and bone lead levels among eligible nonparticipants and participants at baseline. At baseline, 8% were current smokers, 6% were classified as diabetic, 26% were classified as hypertensive, 7% reported using diuretic medication, and 78% reported using analgesic medication. Only 5% of the study subjects had reduced renal function (SCr > 1.5 mg/dL) at baseline. Subjects with diabetes at baseline had slightly higher bone lead levels and lower blood lead levels than those who were free of diabetes. Furthermore, subjects with diabetes had greater increase in SCr over time compared with those free of diabetes at baseline (*p* = 0.03 from Wilcoxon rank-sum test).

The nonparametric Wilcoxon signed-rank test showed that the mean follow-up SCr (1.06 mg/dL) was significantly lower than the mean baseline SCr (1.25 mg/dL), and blood lead levels decreased significantly over time in this population (*p* < 0.05).

### Associations of baseline and follow-up SCr with blood and bone lead levels.

Both bone lead measures, but not blood lead, were consistently and positively associated with baseline SCr in cross-sectional analyses, but these associations were not statistically significant (Table 2). However, a significant interaction between diabetes and baseline tibia lead level regressed on baseline SCr was observed after adjusting for potential confounders (Table 3). Specifically, the positive cross-sectional association of tibia lead level with SCr was substantially stronger and statistically significant among diabetics compared with nondiabetics. Similar effect modification by diabetes was found with respect to the association of baseline patella lead with baseline SCr, but the interaction was not significant (Table 3). Exclusion of diuretic medication users or participants with SCr > 1.5 mg/dL did not materially change the observed associations between lead levels and baseline SCr. No significant interaction between hypertensives and baseline lead levels regressed on baseline SCr was observed (Table 3).

Similarly, both baseline tibia lead measurement and follow-up blood lead levels were consistently and positively associated with follow-up SCr, but only the association of follow-up blood lead with follow-up SCr was statistically significant (Table 2). In analogy to the cross-sectional analysis, a significant interaction between diabetes and tibia lead on follow-up SCr was observed (Table 3). Results remained unchanged after diuretic medication users at follow-up were excluded. Exclusion of participants with SCr > 1.5 mg/dL did not materially change the observed associations between baseline bone lead levels and follow-up SCr. However, the association of follow-up blood lead with follow-up SCr and the interaction of blood lead with diabetes in determining follow-up SCr became nonsignificant after we excluded participants with baseline SCr > 1.5 mg/dL. A significant interaction between hypertensive status and follow-up blood lead level regressed on follow-up SCr was observed (Table 3). Specifically, the positive cross-sectional association of blood lead level with SCr was substantially stronger and statistically significant among hypertensives compared with normotensives.

### Association of annual change in SCr with blood and bone lead levels.

**Figure 1:**
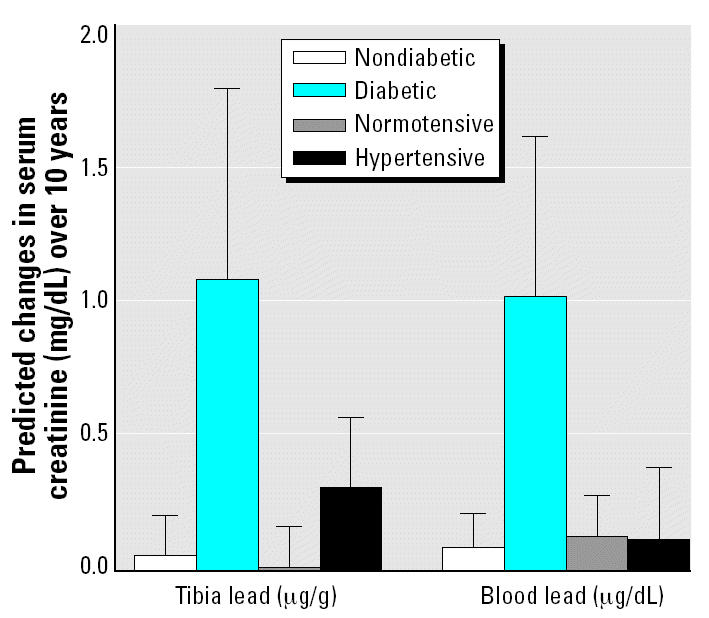
The modifying effect of diabetes and hypertension on the 10-year change in SCr associated with increasing tibia and blood lead levels from the midpoints of their lowest to their highest quartiles (25 μg/g and 8 μg/dL increases, respectively). Error bars indicate 95% confidence intervals.

All three lead measures were positively associated with longitudinal increases in SCr, but none of these associations was statistically significant (Table 2). However, we observed significant interactions of both blood and tibia lead with diabetes in predicting annual change in SCr after adjusting for baseline covariates (Table 3). For example, increasing the tibia lead level from the midpoints of the lowest to the highest quartiles (9–34 μg/g) was associated with an increase in the rate of rise of SCr that was 17.6-fold greater in diabetics than nondiabetics (1.084 mg/dL over 10 years vs. 0.062 mg/dL over 10 years). Similarly, increasing baseline blood lead levels from the midpoints of the lowest to the highest quartiles (3–11.25 μg/dL) was associated with an increase in the rate of rise of SCr that was 12.8-fold greater in diabetics than nondiabetics (1.01 μg /dL over 10 years vs. 0.08 μg /dL over 10 years) (Figure 1). Exclusion of participants with baseline SCr > 1.5 mg/dL did not materially change the observed longitudinal associations between lead levels and change in SCr. The direction of the observed longitudinal associations between lead levels and SCr remained the same after we excluded diuretic medication users at baseline, but the interaction between blood lead and tibia lead and diabetes became nonsignificant. Similar findings were observed after exclusion of hypertensive subjects at baseline.

We also observed significant interactions of tibia lead with hypertension in predicting annual change in SCr after adjusting for baseline covariates (Table 3). Increasing the tibia lead level from the midpoints of the lowest to the highest quartiles (9–34 μg/g) was associated with an increase in the rate of rise of SCr that was > 50-fold greater in hypertensives than in normotensives (0.31 mg/dL over 10 years vs. 0.005 mg/dL over 10 years) (Figure 1).

There was no interaction of alcohol consumption or smoking with lead biomarkers in determining annual change in SCr. Assessment for a potential interaction between race and lead exposure in determining annual change in SCr was limited by small numbers (*n* = 12, 2.7% black participants). Excluding this group from the analysis did not change the observed associations

## Discussion

In this study, significant associations of bone lead (particularly tibia bone) with prospective follow-up measures and annual change in SCr were observed among subjects with diabetes. We also observed significant positive associations of blood lead with prospective annual change in SCr among diabetics and cross-sectional increases in SCr (at the follow-up exam) among nondiabetics. Associations of higher blood lead with poorer renal function have been described elsewhere among non-occupationally exposed populations. A positive correlation between SCr concentration and blood lead levels was found in a survey of men in the British civil service (Staessen et al. 1990). In general population studies in Belgium and in the United States (the NAS cohort in Boston), creatinine clearance was inversely associated with blood lead levels (Payton et al. 1994; Staessen et al. 1992). Furthermore, in a recent longitudinal analysis of the NAS cohort, there was a positive association between low lead levels and SCr (Kim et al. 1996).

The analysis of lead, hypertension, and SCr indicates that both the association between follow-up blood lead with follow-up measures of SCr and the association between tibia lead and prospective annual change in SCr were significantly modified by hypertensive status, with hypertensive subjects having stronger and more significant associations. A recent analysis from the Third National Health and Nutrition Examination Survey (NHANES III) also showed a significant association of higher blood lead levels with chronic kidney disease and elevated SCr among hypertensives. Relationships among lead exposure, impaired renal function, and hypertension are complex: Lead exposure has been associated with an increased risk of hypertension, and essential hypertension, in turn, is a well-established risk factor for kidney disease. Whether lead affects blood pressure indirectly through alterations in kidney function or via more direct effects on the vasculature or neurologic blood pressure control is unknown. The interaction of hypertension, lead, and kidney function merits further investigation in a prospective cohort.

Studies of lead body burden estimated by EDTA mobilization tests have revealed a correlation of high body lead burden with declines in renal function (Batuman et al. 1983; Lin and Huang 1994; Lin et al. 2001). There have been a few studies of the association between body burden in the form of bone lead and renal function, but the results have been inconclusive. Furthermore, most prior studies have assessed occupationally exposed populations. For example, no adverse effects of bone lead on renal function were found in Swedish smelter works (Gerhardsson et al. 1992), whereas a positive association of tibia lead with glomerular hyperfiltration was reported in Belgian lead workers, suggesting the potential for a paradoxical protective effect of bone lead on renal function (Roels et al. 1994). The present study is among the first to assess the relation of bone lead levels from a general population sample with measures of renal function. Our findings support the hypothesis that long-term low-level lead accumulation (estimated by tibia bone lead) is associated with an increased risk of declining renal function particularly among diabetics or hypertensives, populations already at risk for impaired renal function.

However, there are several limitations to our findings. In the absence of diabetes or hypertension, we did not see statistically significant associations of bone lead levels with either cross-sectional or longitudinal measures of renal function. Our study population was not occupationally exposed and therefore had relatively low lead levels, whereas the clearest associations of lead with decrements in renal function have been demonstrated among heavily exposed populations. Although SCr is a widely used measure of renal function in clinical medicine, it provides only a rough estimate of glomerular function. Increases in SCr are relatively insensitive to declining glomerular filtration and are evident (i.e., > 1.5 mg/dL) only when kidney function has been reduced by about 50%. Therefore, low exposures and the relative insensitivity of our outcome measure may have limited our ability to detect more modest lead effects. Furthermore, we observed an unexpected overall decline in SCr over time in this population. SCr is a function of muscle mass and diet, as well as the glomerular filtration rate. A possible explanation for the lower follow-up SCr we observed includes decreased creatinine generation attributable to reduced muscle mass as a result of aging or reduced meat intake. However, SCr level was not associated with total energy-adjusted protein intake either at baseline or at the follow-up in the present study. Therefore, protein intake did not appear to confound the relation of SCr with lead exposure. Baseline and follow-up SCr were measured using the same technique and established standards and calibration methods, making measurement drift an unlikely explanation for lower follow-up values.

In addition, our diagnostic criteria for diabetes may misclassify individuals. However, this type of misclassification is likely to be non-differential with respect to the null hypothesis of no association, because nondiabetic individuals who had high or low lead exposure (and high or low SCr) would be equally likely to be misclassified as diabetic. The same is true regarding diabetic individuals being misclassified as nondiabetics. Such a nondifferential misclassification will tend to drive the overall effect toward a null finding (attenuated parameter estimates) but will not drive a true null finding toward an effect.

Our findings do not necessarily exclude the alternative hypothesis that elevated bone (or blood) lead levels were a result of impaired renal function. However, studies have shown that body lead burden was not elevated among patients with renal insufficiency or chronic renal failure if they did not have a history of childhood plumbism or high lead exposure (Batuman et al. 1983; Lin and Huang 1994; Sanchez-Fructuoso et al. 1996). For the most part, participants’ SCr levels were well within the normal range throughout the follow-up period of the study, and excluding individuals with elevated SCr at baseline did not materially alter our findings. These observations in combination with the prospective study design support the conclusion that the direction of the association is lead dose resulting in renal dysfunction. Hypertensive status has been shown to be associated with increases in SCr (Staessen et al. 1990, 1992) and with bone lead levels in the NAS (Hu et al. 1996). However, inclusion or exclusion of hypertension in the models did not make substantial differences in the observed associations except as noted in the interaction analyses.

Although tibia lead was clearly associated with longitudinal decrements in renal function among the study’s diabetics, patella lead was not. Differential sensitivity of tibia versus patella lead in predicting health outcomes has been observed previously (Payton et al. 1998) and may be a consequence of presumed different lead toxicokinetics in cortical (tibia) and trabecular (patella) bone. Previous research demonstrated overall declines in patella lead but stable tibia lead levels in this population (Kim et al. 1997) during 3 years of follow-up, which implies that patella lead may not reflect past cumulative exposures as accurately as tibia lead levels. In addition, the null finding for patella lead may be due to higher uncertainties, that is, greater measurement error, in patella lead measurements than in tibia lead measurements (Hu et al. 1991).

Several factors related to blood and bone lead levels, including age, cigarette smoking, and alcohol consumption are potential confounders of the lead–SCr relationship. However, SCr level was not associated with age, cigarette smoking, or alcohol use in the present study. Therefore, these factors did not appear to confound the relation of SCr with lead exposure.

In summary, our findings suggest that both blood lead and cumulative lead burden, reflected by tibia (cortical) bone lead levels, are predictors of prospective increases in SCr among middle-age and elderly men with diabetes or hypertension. To our knowledge, no previous studies have reported an analysis of the potential for diabetes to modify the relationship between lead exposure and renal function. Such an interaction may be related to the joint effect of the glomerular pathology associated with diabetes and the tubular atrophy and interstitial nephritis/fibrosis associated with lead. Given how common a history of environmental or occupational lead exposure is among adults and the high prevalence (and growing incidence) of type 2 diabetes in the general population, an interaction as suggested in this study would be of significant public health importance if confirmed. Additional research in this area—both epidemiologic and experimental involving, for example, the diabetic rat—would be helpful.

## Figures and Tables

**Table 1:** Characteristics of the 707 eligible subjects and the 448 NAS subjects at baseline (1991–1995) and at follow-up visit [mean ± SD or no. (%)].

		Participants in this study		
			Follow-up	
Characteristic	Baseline eligible subjects Mean ± SD*^a^*	Baseline Mean ± SD*^a^*	No.	Mean ± SD*^a^*
Tibia lead (μg/g)	21.9 ± 13.3	21.5 ± 13.5	247	23.8 ± 16.8*
Patella lead (μg/g)	32.0 ± 19.6	32.4 ± 20.5	258	31.1 ± 23.5*
Blood lead (μg/dL)	6.2 ± 4.1	6.5 ± 4.2	427	4.5 ± 2.5*
Age (years)	66.9 ± 7.2	66.0 ± 6.6	448	72.0 ± 6.5*
SCr (mg/dL)	1.2 ± 0.2	1.25 ± 0.2	448	1.1 ± 0.4*
Body mass index (kg/m^2^)	27.8 ± 3.8	27.8 ± 3.7	399	28.2 ± 3.9*
Alcohol consumption (g/day)	13.1 ± 17.9	13.4 ± 17.9	386	13.5 ± 20.2
Serum albumin (g/dL)	4.7 ± 0.3	4.7 ± 0.3	448	4.4 ± 0.3*
Pack-years of smoking*^b^*	22.3 ± 25.6	19.5 ± 23.6	437	19.7 ± 24.2*
Energy-adjusted protein intake (g/day)	82.2 ± 15.7	82.4 ± 16.4	386	80.5 ± 15.4*
Follow-up time (year)	ND	ND	448	6.0 ± 0.5
Changes in SCr (mg/dL-year)	ND	ND	448	−0.03 ± 0.1
Smoking status			443	
Never	210 (29.7)*^c^*	145 (32.4)*^c^*		145 (32.7)*^c^*
Current	61 (8.6)	36 (8.0)		26 (5.9)
Former	436 (61.7)	267 (59.6)		272 (61.4)
Hypertensives*^d^*	198 (28.0)	115 (25.7)	448	126 (28.1)
Clinical diagnosed diabetes mellitus	54 (7.6)	26 (5.8)	448	52 (11.6)
Use of diuretics (yes)	62 (8.8)	33 (7.4)	448	63 (14.1)
Use of aspirin or pain medication (yes)	540 (76.4)	349 (77.9)	448	355 (79.2)
SCr > 1.5 mg/dL (yes)	41 (5.8)	24 (5.4)	448	23 (5.1)
Alcohol consumption			386	
None	184 (26.1)	108 (24.1)		94 (24.4)
0–20 (g/day)	371 (52.5)	243 (54.2)		215 (55.7)
≥ 20 (g/day)	152 (21.5)	97 (21.7)		77 (20.0)

ND, no data.

a Values are mean ± SD except where indicated.

b Nine eligible subjects and six baseline subjects were missing pack-year smoking data.

c No. (%).

d Hypertensive was defined as systolic blood pressure ≥ 160, or diastolic blood pressure ≥ 95 mmHg, or a physician’s diagnosis of hypertension with use of antihypertensive medication.

* Values at baseline and follow-up were significantly different (*p* < 0.05 by Wilcoxon signed-rank test).

**Table 2:** Multiple regression analysis of SCr on blood or bone lead in the NAS [β (SE)].

Variable	Model of baseline SCr*^a^*	Model of follow-up SCr*^b^*	Model of changes in SCr*^c^*
Log_e_(baseline blood lead)	−0.023 (0.019)		0.009 (0.005)
Log_e_(follow-up blood lead)		0.149 (0.055)*	
Log_e_(baseline patella lead)	0.011 (0.017)	−0.006 (0.043)	0.001 (0.004)
Log_e_(baseline tibia lead)	0.017 (0.020)	0.065 (0.049)	0.007 (0.005)

a Adjusted for age, age squared, BMI, alcohol intake (< 20, ≥ 20 g/day vs. nondrinkers), ever smoking, pain medication, hypertension, and diabetes.

b Adjusted for follow-up variables of age, BMI, alcohol intake (< 20, ≥ 20 g/day vs. non-drinkers), ever smoking, pain medication, hypertension, and diabetes.

c Adjusted for baseline variables of SCr, SCr squared, age, BMI, alcohol intake (< 20, ≥ 20 g/day vs. nondrinkers), ever smoking, pain medication, hypertension, and diabetes.

* *p* < 0.05 for the -coefficient.

**Table 3:** Multiple regression analysis of SCr on blood or bone lead in the NAS stratified by baseline diabetic status [β (SE)].

Variable, diabetic or hypertensive status	Model of baseline SCr*^a^*	Model of follow-up SCr*^b^*	Model of changes in SCr*^c^*
Log_e_ (baseline blood lead)			
Diabetic (*n* = 26)	−0.054 (0.089)		0.076 (0.023)*^,^**
Nondiabetic (*n* = 422)	−0.022 (0.019)		0.006 (0.005)
Hypertensive (*n* = 115)	−0.009 (0.039)		0.008 (0.010)
Normotensive (*n* = 333)	−0.027 (0.021)		0.009 (0.006)
Log_e_ (follow-up blood lead)			
Diabetic (*n* = 24)		0.223 (0.183)	
Nondiabetic (*n* = 403)		0.142 (0.058)*	
Hypertensive (*n* = 108)		0.352 (0.097)*^,^**	
Normotensive (*n* = 319)		0.058 (0.065)	
Log_e_ (baseline patella lead)			
Diabetic (*n* = 26)	0.056 (0.065)	0.007 (0.107)	0.004 (0.017)
Nondiabetic (*n* = 422)	0.008 (0.017)	−0.008 (0.047)	0.0004 (0.005)
Hypertensive (*n* = 115)	0.052 (0.034)	−0.019 (0.075)	0.009 (0.009)
Normotensive (*n* = 333)	−0.0003 (0.019)	−0.0005 (0.051)	−0.002 (0.005)
Log_e_ (baseline tibia lead)			
Diabetic (*n* = 26)	0.229 (0.102)*^,^**	0.699 (0.192)*^,^**	0.082 (0.027)*^,^**
Nondiabetic (*n* = 422)	0.011 (0.020)	0.029 (0.049)	0.005 (0.005)
Hypertensive (*n* = 115)	0.027 (0.037)	0.180 (0.097)	0.023 (0.010)*^,^**
Normotensive (*n* = 333)	0.013 (0.024)	0.030 (0.055)	0.0004 (0.006)

a Adjusted for age, age squared, BMI, alcohol intake (< 20, ≥ 20 g/day vs. nondrinkers), ever smoking, pain medication, hypertension, and diabetes.

b Adjusted for follow-up variables of age, BMI, alcohol intake (< 20, ≥ 20 g/day vs.nondrinkers), ever smoking, pain medication, hypertension, and diabetes.

c Adjusted for baseline variables of SCr, SCr squared, age, BMI, alcohol intake (< 20, ≥ 20 g/day vs. nondrinkers), ever smoking, pain medication, hypertension, and diabetes.

* *p* < 0.05 for the β-coefficient.

** *p* < 0.05 for the interaction between lead variable and diabetic or hypertensive status.
